# TnP as a Multifaceted Therapeutic Peptide with System-Wide Regulatory Capacity

**DOI:** 10.3390/ph18081146

**Published:** 2025-08-01

**Authors:** Geonildo Rodrigo Disner, Emma Wincent, Carla Lima, Monica Lopes-Ferreira

**Affiliations:** 1 Plataforma Zebrafish of the Laboratory of Applied Toxinology (CeTICS/FAPESP), Butantan Institute, São Paulo 05503-900, Brazil; geonildo.disner.esib@esib.butantan.gov.br (G.R.D.); carla.lima@butantan.gov.br (C.L.); 2 Unit of Systems Toxicology, Institute of Environmental Medicine, Karolinska Institutet, 171 77 Solna, Sweden

**Keywords:** TnP, therapeutic peptide, zebrafish, network pharmacology, drug metabolism, wound healing, proteostasis

## Abstract

**Background:** The candidate therapeutic peptide TnP demonstrates broad, system-level regulatory capacity, revealed through integrated network analysis from transcriptomic data in zebrafish. Our study primarily identifies TnP as a multifaceted modulator of drug metabolism, wound healing, proteolytic activity, and pigmentation pathways. **Results:** Transcriptomic profiling of TnP-treated larvae following tail fin amputation revealed 558 differentially expressed genes (DEGs), categorized into four functional networks: (1) drug-metabolizing enzymes (*cyp3a65*, *cyp1a*) and transporters (SLC/ABC families), where TnP alters xenobiotic processing through Phase I/II modulation; (2) cellular trafficking and immune regulation, with upregulated myosin genes (*myhb*/*mylz3*) enhancing wound repair and *tlr5*-*cdc42* signaling fine-tuning inflammation; (3) proteolytic cascades (*c6ast4*, *prss1*) coupled to autophagy (*ulk1a*, *atg2a*) and metabolic rewiring (*g6pca.1*-*tg* axis); and (4) melanogenesis-circadian networks (*pmela*/*dct*-*fbxl3l*) linked to ubiquitin-mediated protein turnover. Key findings highlight TnP’s unique coordination of rapid (protease activation) and sustained (metabolic adaptation) responses, enabled by short network path lengths (1.6–2.1 edges). Hub genes, such as *nr1i2* (*pxr*), *ppara*, and *bcl6aa/b*, mediate crosstalk between these systems, while potential risks—including muscle hypercontractility (*myhb* overexpression) or cardiovascular effects (*ace2*-*ppp3ccb*)—underscore the need for targeted delivery. The zebrafish model validated TnP-conserved mechanisms with human relevance, particularly in drug metabolism and tissue repair. TnP’s ability to synchronize extracellular matrix remodeling, immune resolution, and metabolic homeostasis supports its development for the treatment of fibrosis, metabolic disorders, and inflammatory conditions. **Conclusions:** Future work should focus on optimizing tissue-specific delivery and assessing genetic variability to advance clinical translation. This system-level analysis positions TnP as a model example for next-generation multi-pathway therapeutics.

## 1. Introduction

Existing anti-inflammatory therapies are limited to steroidal and non-steroidal anti-inflammatory agents. Sadly, chronic use of these drugs is reported to allegedly cause severe adverse effects like gastrointestinal, cardiovascular, and renal abnormalities [1]. In this context, there is a critical need to discover new anti-inflammatory agents with selective action, higher efficacy, and reduced toxicity. Not only does the complexity of different inflammatory processes pose a challenge for pharmaceutical chemists, but the identification of new compounds with desirable activity and pharmacokinetic properties remains a core setback in the new drug development pipeline [1,2].

Over the last few years, naturally sourced molecules or natural compounds have emerged as a significant source of drug candidates being evaluated for their anti-inflammatory actions [1]. Typically, natural products are considered safe, efficacious, biocompatible, and cost-effective alternatives for treating inflammatory diseases, and developing derived drugs is a rational and productive strategy [3]. Still, toxin-based molecules and animal toxin-derived peptides are promising and interesting sources of new anti-inflammatories, which have been widely explored. Notably, peptides from microorganisms, plants, and animals have been suggested as having superior therapeutic properties compared to human counterparts, which include higher potency, selectivity, and in vivo stability, gaining considerable interest as potential therapeutic options for inflammatory conditions [4,5].

Within this framework, our group has been working with the TnP family of synthetic cyclic peptides, first discovered in the venom of the Brazilian fish *Thalassophryne nattereri*. TnP has great potential to offer an alternative therapy to inflammatory conditions, is patented in several countries, and has had its therapeutic potential confirmed in murine models of experimental autoimmune encephalomyelitis (EAE) for multiple sclerosis (MS) and asthma as well as miRNA-mediated neutrophilia control in tail-amputated zebrafish challenged with lipopolysaccharide (LPS) [6,7,8].

Among the various immunomodulatory effects of TnP, its role in regulating the entry of inflammatory cells, including macrophages, Th1, and Th17 lymphocytes, into the central nervous system (CNS) is noteworthy [9]. Additionally, it inhibits the expression of α4β1 integrin receptors on the blood-brain barrier, a crucial event that precedes perivascular infiltration and disease onset [6].

Using zebrafish as a preclinical toxicology model, we demonstrated a broad therapeutic index, with non-lethal doses ranging from 1 nM to 10 μM and no observed neurotoxicity or cardiotoxicity, supporting TnP’s potential as a promising therapeutic candidate [10].

Omics-based approaches, such as genome-wide transcriptional profiling, offer a powerful strategy to address current limitations in drug screening by enabling comprehensive analysis of drug-induced gene expression changes [11]. Transcriptomics offers a comprehensive view of molecular alterations by quantifying ribonucleic acid (RNA) transcripts across the entire genome at a given time, thereby revealing drug-responsive gene expression patterns [12]. This approach aids in target identification (proteins physically binding to the drug or to proteins that are only functionally related), efficacy and toxicity evaluation, and optimization of dosing regimens.

In this study, we aimed to assess the molecular clues of the therapeutic anti-inflammatory effect of TnP during early signaling in a zebrafish wound-injury model, using tail fin amputation. To achieve this, we performed an integrative gene expression analysis across transcriptomic profiling datasets from TnP-treated tail-fin-amputated zebrafish. The differentially expressed genes (DEGs) were then mapped to the STRING on Cytoscape database to construct protein-protein interaction (PPI) networks. Functional clusters within these networks were subsequently identified.

## 2. Results

### 2.1. TnP Significantly Impacts Drug Metabolism Networks

One of the most crucial setbacks in today’s scientific process, particularly in drug development programs, is the need for efficient experimental models. These models typically require elevated costs, are time-consuming, and yield limited success rates. Although the use of alternative approaches to animal experimentation has been increasing in the last decade, to advance the drug discovery pipeline, animal experimentation is still vital. For this reason, the vertebrate zebrafish model is a promising option due to its well-known characteristics, such as high genetic similarity to humans, rapid development, high reproducibility, and lower cost compared to traditional rodent counterparts. Additionally, it offers versatility in drug screening, enabling toxicity assessment and large-scale effectiveness. Zebrafish have been efficiently used in high-throughput screening, safety assessment (e.g., cardiac toxicity, hepatic toxicity, and neurotoxicity), validation of therapeutic targets, and studies of mechanisms of action [13,14].

Not only showing efficacy but also understanding the mechanism of action within a living whole-animal system is one of the essential barriers to drug discovery [15]. Often, several compounds have activity in the first stages of study, i.e., in vitro or cell systems; unfortunately, they do not always represent a similar context for an organ or tissue within the body. There are examples of some drugs that are effective in humans but not in zebrafish, and vice versa. Nevertheless, over 20 years of drug screening in zebrafish suggest that, in general, molecules active in zebrafish exhibit similar activity in mice and humans, with comparable pharmacokinetic properties [16].

For certain drugs, zebrafish replicate human responses even more accurately than mouse models. Thalidomide, for example, caused thousands of congenital disabilities in unborn children. In mice, it did not induce any defects. On the other hand, in zebrafish, thalidomide triggered morphological limb defects similar to those in humans [15].

Remarkably, the use of zebrafish for drug screening and pharmacological studies has received increasing attention in the fields of drug absorption, distribution, metabolism, and excretion (ADME) because of the similarity of drug metabolism between zebrafish and mammals [17,18,19].

Therapeutic peptides are a class of pharmaceutical agents made from chains of amino acids used to treat various conditions. This type of medicine can be engineered or isolated from natural sources and then chemically synthesized. Therefore, peptide drug development has emerged as a prominent topic in pharmaceutical research. There are many examples of peptide drugs; insulin, for example, is the first commercial peptide drug and has been thoroughly used to treat diabetes for many years. This class of drugs is distinguished by their ability to have multiple therapeutic effects, as they target specific receptors by mimicking natural signaling molecules (e.g., hormones, cytokines), which can lead to diverse physiological responses [20]. Also, they may play multiple roles, serving as both anti-inflammatory agents and immune modulators. Importantly, peptides function in immune system modulation, making them effective in treating multiple conditions.

In this context, TnP is a drug candidate with considerable therapeutic potential. Besides its beneficial effects in mice, previous results suggest cellular activation after treatment, notably through the Aryl Hydrocarbon Receptor-Cytochrome P450 (AHR-CYP) axis in zebrafish. In order to understand the molecular signature of the therapeutic effect of the TnP treatment in the inflammation model based on tail fin amputation, we performed the whole sequencing of the transcriptome in zebrafish larvae, identifying that TnP exposure altered the molecular response to inflammation by modulating genes related to acute neutrophil infiltration, among others [21].

The accumulation of gene transcriptional profiling data provides us with an unprecedented opportunity to explore the common specific pathways involved in TnP’s anti-inflammatory effect at the system level. Using an injured larvae-treated model (100 mM TnP), we identified 558 DEGs compared to untreated control.

From this set, we grouped the DEGs into four expert-selected categories: (I) drug metabolizing enzymes, (II) cellular traffic, (III) cell activity, and (IV) signaling pathways. These subsets were used to construct the corresponding PPI networks using STRING in Cytoscape, although the same genes can participate in different molecular processes in parallel. The final list ended with the following number of genes regulated by TnP in each class: in I, 74 were upregulated and 24 downregulated; in II, 80 were upregulated and 25 downregulated; in III, 57 were upregulated and 49 downregulated; and in IV, 66 were upregulated and 80 downregulated, as reported in Supplementary Table S1. Genes that remained unannotated or did not align with the defined major categories were not included.

Drug perturbation can be observed through differentially expressed (i.e., deregulated) genes located near drug targets in the network topology. The association between deregulated genes and drug targets can be assessed by analyzing shortest-path distances in functional interaction networks, where affected genes may represent either direct interactors or proximal neighbors of the drug target [22].

Once a drug is administered to zebrafish, it undergoes a series of enzymatic reactions driven by drug-metabolizing enzymes (DMEs)—a diverse group responsible for the biotransformation of endogenous and exogenous compounds. These reactions are categorized primarily into Phase I (functionalization) and Phase II (conjugation) metabolism, mirroring the hepatic metabolism pathways observed in humans [23].

Phase I reactions, predominantly catabolic, involve oxidation, reduction, or hydrolysis of drug compounds [23]. The CYP family plays a central role in these reactions, acting as the primary catalysts and regulators of metabolic clearance [24]. Zebrafish possess a complete set of CYP enzymes, many of which share high sequence homology with their human homologs involved in endogenous metabolism. However, xenobiotic-metabolizing CYPs show greater interspecies variability, meaning some drugs may be processed differently in zebrafish compared to humans [19,25].

Phase II reactions, in contrast, are anabolic and involve conjugation—the addition of hydrophilic groups to enhance excretion. Key enzymes in this phase include UDP-glucuronosyltransferases (UGTs), glutathione S-transferases (GSTs), sulfotransferases (SULTs), methyltransferases, and N-acetyltransferases (NATs) [24,26]. These pathways further demonstrate the functional conservation between zebrafish and human drug metabolism, reinforcing the zebrafish as a valuable model for pharmacological studies.

**Figure 1:**
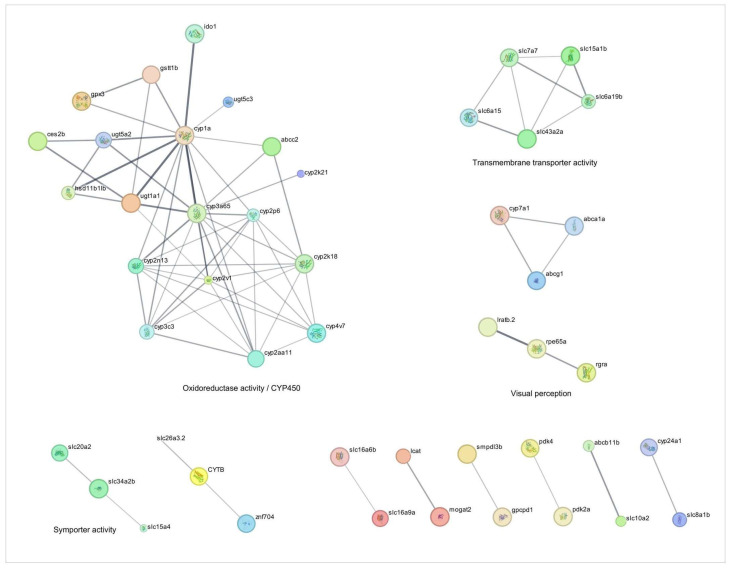
Network analysis of drug-metabolizing enzymes-related genes overexpressed during injury-induced inflammation in zebrafish larvae treated with TnP compared to untreated control. Protein-protein interaction (PPI) networks of 74 differentially overexpressed genes (*p*_adj_ < 0.05). The PPI network was performed in STRING on Cytoscape, and each subnetwork was evaluated in the software to provide the main functional enrichment term through STRING. The bigger the circle, the lower the adjusted *p*-value. Singletons were omitted from the network.

**Figure 2:**
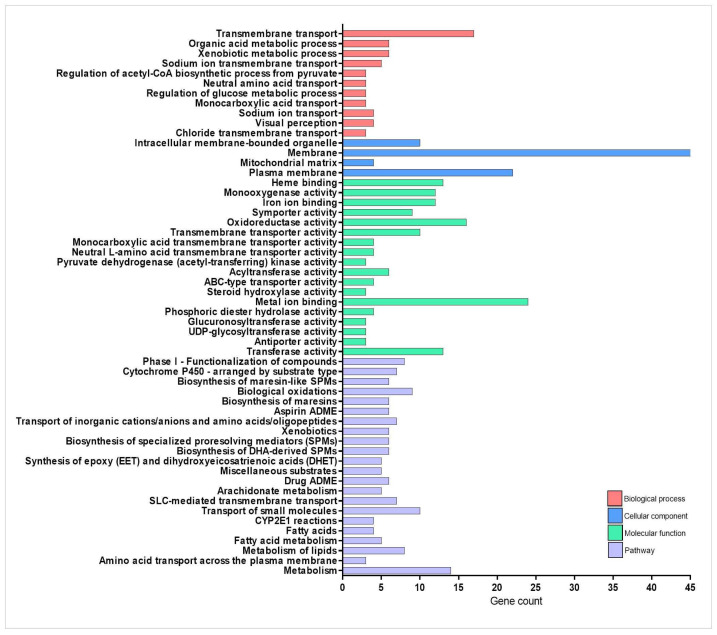
Enrichment analysis of drug-metabolizing enzymes-related genes overexpressed during injury-induced inflammation in zebrafish larvae treated with TnP compared to untreated control. Functional enrichment analysis focusing on the overexpressed genes list (71 DAVID IDs) related to drug-metabolizing enzymes using the DAVID software. Terms were considered significant if the adjusted *p*-value was less than 0.05 and the gene count at least 3.

Regarding the drug metabolism process, the intrayolk administration of the TnP the day before the injury-induced inflammation activated the classic metabolizing pathways (Figure 1 and Figure 2 and Table 1). Our integrated analysis of genes deregulated by TnP reveals its extensive influence on biological networks crucial for drug metabolism and transport. As shown in Figure 1, TnP treatment significantly perturbs functional networks through direct and indirect mechanisms, with important implications for pharmacological interactions.

“Cytochrome P450” and “solute carrier (SLC)-mediated transport” had the largest gene counts, implying that TnP strongly modulates these pathways. The network topology analysis demonstrates that TnP-upregulated genes are predominantly involved in drug metabolism (including key genes such as *cyp3a65*, *cyp2k18*, *cyp1a*, *cyp2n13*, and *cyp7a1*) and gatekeepers of cellular flux (particularly transporters like *slc7a7*, *slc15a1b*, *slc16a6b*, *abcc2*, and *abcb11b*). These genes show direct connectivity (shortest-path distances ≤ 1) to known drug targets, suggesting TnP may substantially alter drug metabolism and distribution. For instance, these CYP enzymes drive Phase I xenobiotic metabolism, oxidizing drugs and toxins to prepare them for elimination. *Cyp3a65* and *cyp2k18* are critical in drug ADME. As highlighted in Figure 1, these genes collaborate with UGTs (e.g., *ugt1a1*, *ugt5a2*) for Phase II conjugation, forming a detoxification cascade, and *cyp7a1* bridges drug metabolism and lipid homeostasis by initiating bile acid synthesis. Particularly, *abcc2* and *abcb11b* export drug metabolites, influencing bioavailability and toxicity. *Slc7a7* and *slc15a1b* mediate amino acid uptake, and *slc16a6b* transports monocarboxylates, supporting energy metabolism.

Key hub genes with high network centrality (degree centrality ≥ 10) emerge as critical regulators of TnP effects. These include UGTs, such as *ugt1a1*, *ugt5a2*, and *ugt5c3*, which are responsible for catalyzing glucuronidation, rendering lipophilic compounds water-soluble for excretion. Their synergy with CYPs, for example, occurs through the generation of metabolites by *cyp3a65*, which are further processed by *ugt1a1*.

The map of the TnP effect illustrates the interaction between *cyp7a1* and *slc10a2* (bile acid transporter) in bile acid synthesis. Bidirectional interaction between *lcat* (esterifies cholesterol) and *abca1a* (mediates cholesterol efflux) regulates cholesterol. Disruption of these players may alter lipid profiles and bile acid signaling.

TnP induced other genes, such as *gstt1b*, *ido1*, and specifically *gpx3* (an antioxidant), playing a role in mitigating oxidative stress induced by CYP activity. Meanwhile, *ido1* modulates inflammation via tryptophan metabolism, potentially linking TnP to immune regulation.

Conversely, TnP downregulates genes involved in fatty acid metabolism, which may reflect compensatory mechanisms or secondary metabolic effects. The biological and clinical implications of this analysis suggest that TnP’s dysregulation of *cyp3a65* or *abcc2* could elevate toxicity or reduce the efficacy of co-administered drugs (e.g., statins, antifungals). Altered *cyp7a1* or *lcat* activity may perturb cholesterol homeostasis, and the *gpx3*/*gstt1b* hubs suggest that TnP influences redox balance, with implications for cytotoxicity. This dual regulation pattern indicates that TnP simultaneously activates detoxification pathways while suppressing specific metabolic processes.

The upregulated gene network analysis reveals that TnP induces broad transcriptional reprogramming, primarily targeting drug-processing hubs (CYPs, UGTs, and SLC/ABC transporters)—risking drug–drug interactions (DDIs)—, metabolic regulators (*cyp7a1*, *lcat*), potentially disrupting lipid homeostasis, and redox sensors (*gpx3*, *ido1*), implicating TnP in oxidative stress and inflammation modulation.

Our integrated analysis demonstrates that TnP has a significant impact on drug metabolism networks through direct target modulation, core gene-mediated pathway regulation, and broad metabolic reconfiguration.

### 2.2. TnP Promotes Wound Healing Through Multi-Pathway Regulation

In this study, our next objective was to decipher the molecular regulators of immune cell trafficking, thereby characterizing the immunomodulatory potential of the TnP and identifying new therapeutic targets for inflammatory resolution pathways. The temporal resolution of the model allows precise tracking of neutrophils between 0–12 h post injury (hpi) (phase of dominated oxidative burst) and macrophages between 12–48 hpi (cytokine signaling phase). The late phase (48–72 hpi) is characterized by inflammation resolution and tissue remodeling [27,28].

**Figure 3:**
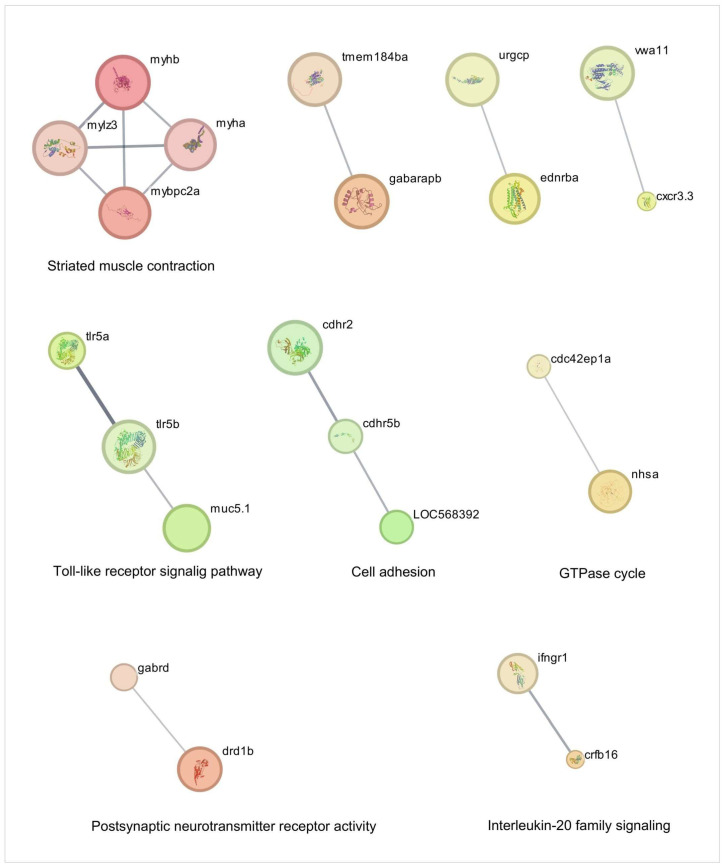
Network analysis of cellular traffic-related genes overexpressed during injury-induced inflammation in zebrafish larvae treated with TnP compared to untreated control. Protein-protein interaction (PPI) networks of 80 differentially overexpressed genes (*p*_adj_ < 0.05). The PPI network was performed in STRING on Cytoscape, and each subnetwork was evaluated in the software to provide the main functional enrichment term through STRING. The bigger the circle, the lower the adjusted *p*-value. Singletons were omitted from the network.

**Figure 4:**
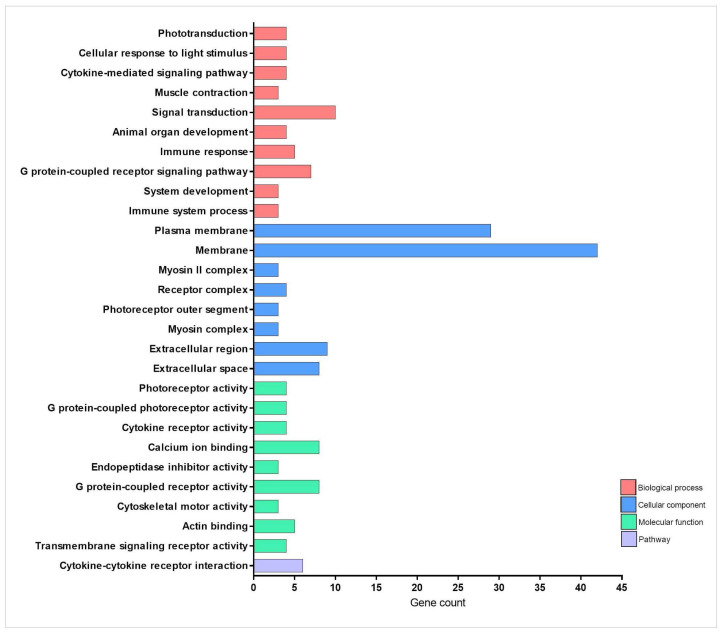
Enrichment analysis of cellular traffic-related genes overexpressed during injury-induced inflammation in zebrafish larvae treated with TnP compared to untreated control. Functional enrichment analysis focusing on the overexpressed genes list (74 DAVID IDs) related to cellular traffic using the DAVID software. Terms were considered significant if the adjusted *p*-value was less than 0.05 and the gene count at least 3.

Our network analysis of TnP effects reveals its multifaceted role in regulating cellular processes through direct interactions (shortest-path distance ≤ 1) with key pathways and genes (Figure 3 and Figure 4 and Table 1). The most prominent impact occurs in striated muscle contraction, where TnP upregulates myosin-related genes (*myhb*, *mylz3*, *myha*, *mybpc2a*) that interact with *tmem184ba* (a membrane protein) and *gabarapb* (a GABA receptor-associated protein), linking muscle function to neuronal signaling. This suggests that TnP modulates cell contractility through myosin-actin dynamics.

Simultaneously, TnP influences innate immunity and immune-inflammatory signaling through two principal mechanisms: *tlr5a*/*tlr5b* (Toll-like receptors—TLR), *cxcr3.3* (chemokine receptor), *ifngr1*, *crfb16*, *cdc42* axis, and mucin-chemokine crosstalk. Genes such as *ifngr1*, *crfb16*, and *cxcr3.3* crosstalk with *tlr5* to promote cytokine release and chemotaxis. Direct connectivity between *tlr5a/b* and the guanosine triphosphatase (GTPase) modulator *cdc42ep1a* likely positions TnP as an immunomodulator, particularly in responses to Gram-negative pathogens. The *muc5.1*-*vwa11* (von Willebrand factor domain)/*cxcr3.3* axis suggests that TnP may regulate neutrophil recruitment, extracellular matrix (ECM) stability, and mucosal immunity.

Notably, TnP targets neuro-immune hubs with high betweenness centrality, including *gabrd* (Gamma-Aminobutyric Acid—GABA receptor), *drd1b* (dopamine receptor), and *ednrba* (endothelin receptor), which are connected to cytokine pathways (*crfb16*, *ifngr1*), implying that TnP bridges neural and immune responses. This could modulate inflammatory tone and leukocyte extravasation, as observed in the endothelial interaction between *ednrba* and *cxcr3.3*. The cadherin-related markers *cdhr2*, *cdhr5b*, *loc568392* (cadherin-like), and *cdc42ep1a* (GTPase regulator) induced by TnP in the interface with GTPase signaling, potentially altering actin cytoskeleton remodeling via *cdc42*, receptor internalization, and cellular trafficking.

TnP’s coordinated regulation of these networks presents both opportunities and challenges. First, TnP could enhance tissue repair via myosin-*cdc42*-driven cell recruitment and balance inflammation through GABA-*tlr5* crosstalk. The potential risks include muscle hypercontractility resulting from *myhb*/*myha* overexpression or mucosal barrier dysfunction due to *muc5.1* disruption.

Overall, TnP emerges as a multi-network modulator that integrates mechanical forces (myosin-actin dynamics), immune coordination (TLR-chemokine signaling), and neuroimmune communication (GABA-dopamine-cytokine axes), potentially enhancing wound healing processes.

### 2.3. TnP Coordinates Proteolytic and Metabolic Pathways

**Figure 5:**
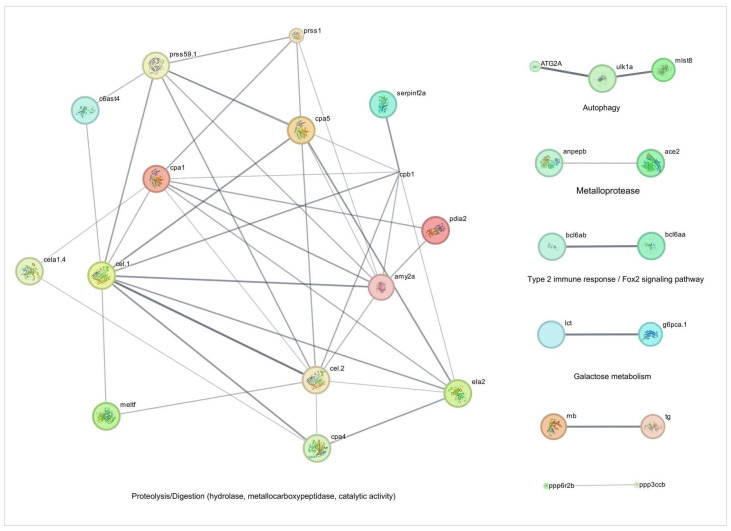
Network and enrichment analysis of cell activity-related genes overexpressed during injury-induced inflammation in zebrafish larvae treated with TnP compared to untreated control. Protein-protein interaction (PPI) networks of 57 differentially overexpressed genes (*p*_adj_ < 0.05). The PPI network was performed in STRING on Cytoscape, and each subnetwork was evaluated in the software to provide the main functional enrichment term through STRING. The bigger the circle, the lower the adjusted *p*-value. Singletons were omitted from the network.

**Figure 6:**
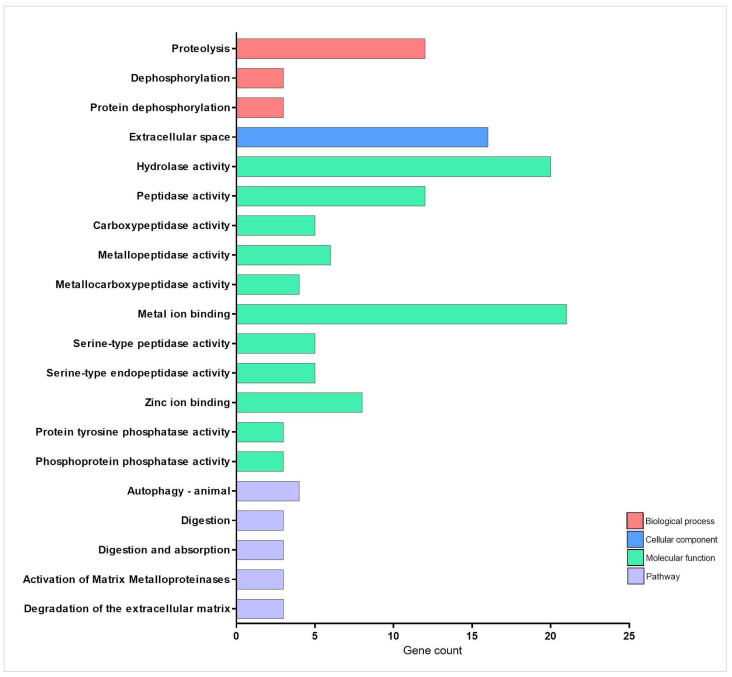
Enrichment analysis of cell activity-related genes overexpressed during injury-induced inflammation in zebrafish larvae treated with TnP compared to untreated control. Functional enrichment analysis focusing on the overexpressed genes list (52 DAVID IDs) related to cell activity using the DAVID software. Terms were considered significant if the adjusted *p*-value was less than 0.05 and the gene count at least 3.

As demonstrated in Figure 5 and Figure 6 and Table 1, TnP coordinates a complex network of proteolytic and metabolic pathways, driving ECM remodeling, intracellular protein turnover, and metabolic adaptation. The network reveals a dominant cluster of metalloproteases, including *c6ast4* (astacin family) and *cpa1*/*cpa4*/*cpa5* (carboxypeptidases), which collectively facilitate ECM degradation, neuropeptide processing, and tissue restructuring. These enzymes interface with serine proteases, such as *prss59.1*, *prss1* (serine proteases), and *ela2*, forming a tightly regulated cascade that balances proteolytic activity through inhibitors like *serpinf2a* and quality control mechanisms involving *pdia2*. This system enables precise control over processes ranging from chronic wound healing to coagulation.

A critical feature of TnP’s action is its integration of proteolysis with autophagy and metabolic signaling. The lipolytic-autophagy axis, involving *cel.1*/*cel.2* (carboxyl ester lipases) and *atg2a*/*ulk1a* (autophagy initiation), demonstrates how lipid breakdown fuels autophagosome formation under mechanistic target of rapamycin (mTOR) regulation by *mlst8*. This coupling ensures efficient cellular clearance while maintaining energy homeostasis. Meanwhile, metabolic crosstalk emerges through unexpected connections, such as the glucose-thyroid axis, where *g6pca.1* (a gluconeogenic regulator) interacts with thyroglobulin (*tg*) processing and lactose metabolism (*lct*), suggesting TnP-mediated metabolic rewiring during fasting or stress.

Transcriptional regulation further refines this system, with *bcl6* paralogs (*bcl6aa/b*) repressing both protease genes (*ela2*) and metabolic enzymes (*lct*), indicating epigenetic control over digestive and degradative functions. Additionally, phosphatase checkpoints, particularly *ppp3ccb* (calcineurin-like) and *ppp6r2b* (PP6 regulatory subunit), modulate muscle energetics (via myoglobin interaction) and autophagy initiation, ensuring precise phosphorylation-dependent signaling.

The network’s efficiency is underscored by its short path length (1.6 edges), enabling rapid, system-wide responses. TnP acts as a biological synchronizer, temporally coordinating the activation of acute proteases with sustained cytoskeletal remodeling and spatially localizing activity to focal adhesions (e.g., *cpa4*-integrin interactions). This capacity for multi-compartment regulation—spanning extracellular (*c6ast4*, *ela2*), membrane-bound (*anpepb*), and intracellular (*atg2a*) processes—positions TnP as a versatile therapeutic candidate. Potential applications include fibrosis treatment via ECM remodeling, metabolic syndrome intervention through *g6pca.1* modulation, and neuro-immune communication tuning via protease-inhibitor balance (*serpinf2a*-*prss1*).

However, the system’s sensitivity necessitates careful optimization to avoid adverse effects, such as excessive tissue degradation from unchecked *ela2* activity or cardiovascular disruptions via *ace2*-*ppp3ccb* signaling. Future studies should prioritize temporal resolution of TnP’s effects and patient-specific modeling to account for genetic variability (e.g., *bcl6* polymorphisms). By harnessing its unique ability to synchronize proteolytic, metabolic, and signaling networks, TnP offers a promising framework for addressing complex diseases that require the coordinated intervention of multiple pathways.

### 2.4. TnP Regulates Pigmentation, Metabolism, and Ubiquitin Signaling

**Figure 7:**
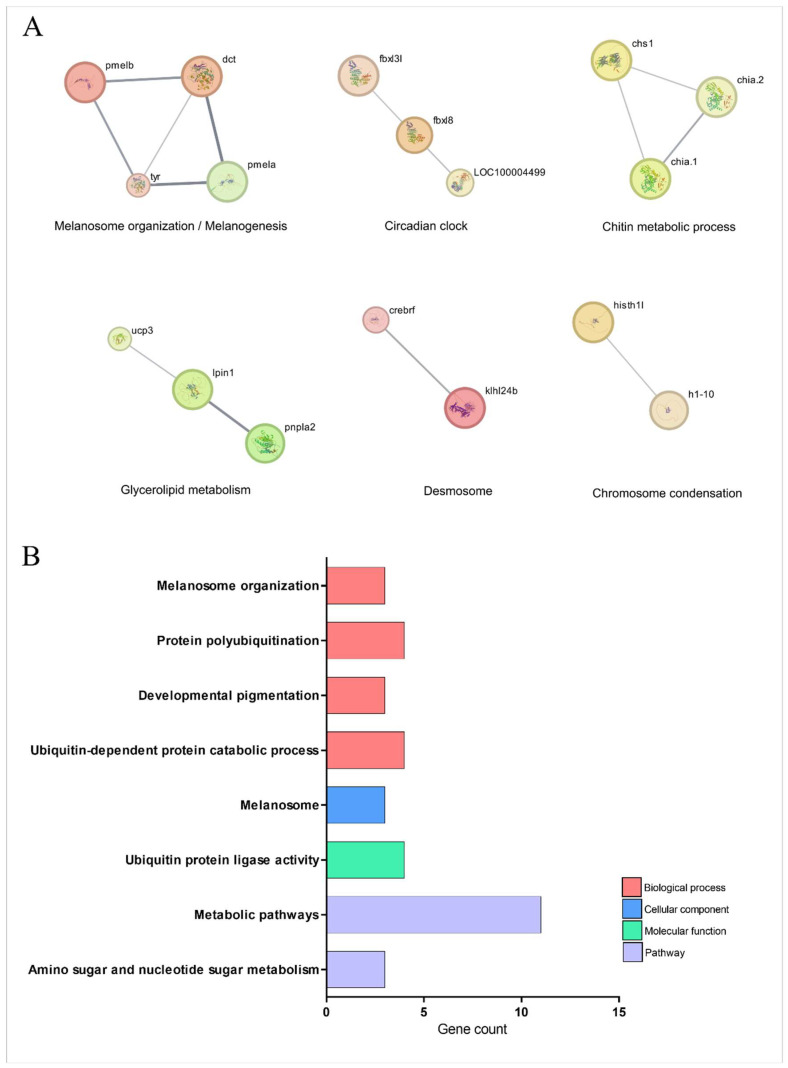
Network and enrichment analysis of signaling pathway-related genes overexpressed during injury-induced inflammation in zebrafish larvae treated with TnP compared to untreated control. (**A**) Protein-protein interaction (PPI) networks of 66 differentially overexpressed genes (*p*_adj_ < 0.05). The PPI network was performed in STRING on Cytoscape, and each subnetwork was evaluated in the software to provide the main functional enrichment term through STRING. The bigger the circle, the lower the adjusted *p*-value. Singletons were omitted from the network. (**B**) Functional enrichment analysis focusing on the overexpressed genes list (62 DAVID IDs) related to signaling pathways using the DAVID software. Terms were considered significant if the adjusted *p*-value was less than 0.05 and the gene count at least 3.

Figure 7 and Table 1 highlights the signaling pathways orchestrated by TnP, which are associated with specific biological processes, including melanogenesis, lipid metabolism, ubiquitin-dependent protein degradation, and chitin metabolism. The analysis reveals strong connectivity between genes involved in pathways related to pigmentation, melanosome organization, and metabolic regulation.

Central to this network is the melanogenic machinery, where structural proteins PMELA/PMELB form the melanosome matrix scaffold alongside enzymatic regulators *dct* and *tyr*, which drive melanin synthesis. This pathway intersects with circadian control through F-box proteins FBXL3L/FBXL8, which link ubiquitin-mediated degradation of clock proteins to melanocyte differentiation rhythms. The system exhibits remarkable coupling between melanosome biogenesis (*pmela*-*pmelb*-*dct* axis), protein turnover (*fbxl3l* ubiquitin ligase activity), and chitin metabolism (*chs1*-*chia.1/2* interaction).

TnP further modulates a metabolic-desmosome network, where lipid homeostasis regulators, such as *ucp3* (mitochondrial uncoupling), *lpin1* (phosphatidic acid phosphatase), and *pnpla2* (adipose triglyceride lipase), unexpectedly interface with structural components. These interactions bridge desmosome maintenance (via *klhl24b*) with chromatin organization (*histh1l-h1-10*), suggesting that TnP influences skin barrier integrity through energy metabolism and nuclear-cytoplasmic signaling in pigment cells.

The ubiquitin-proteasome system emerges as a critical node, with TnP regulating polyubiquitination specialists (*fbxl3l*/*fbxl8*), chaperone-like factors (*crebrf*), and metabolic interfaces (*pnpla2* ubiquitination). Collectively, these findings position TnP as a multi-compartment coordinator that synchronizes circadian protein turnover with melanogenesis, nuclear-cytoplasmic lipid droplet communication, and desmosome integrity, thereby maintaining energy homeostasis.

The network’s short path lengths (average 2.1 edges) enable TnP to regulate rapid ubiquitin-mediated events and sustain metabolic adaptations simultaneously, highlighting its potential as a therapeutic agent for pigmentary, metabolic, and circadian disorders. Future studies should explore tissue-specific delivery mechanisms to optimize these effects while minimizing off-target impacts on cardiac and adipose function.

## 3. Discussion

The comprehensive transcriptomic and network analysis of TnP’s effects in zebrafish reveals, in descending order of importance, its broad influence on drug metabolism, wound healing, proteolytic pathways, and pigmentation/metabolic signaling. These findings highlight TnP as a promising multi-target therapeutic candidate with implications for inflammation resolution, tissue repair, and metabolic regulation.

TnP exerts a profound effect on drug metabolism by modulating cytochrome P450 enzymes, phase II conjugation pathways (UGTs, GSTs), and membrane transporters (SLC/ABC family). The upregulation of key genes (e.g., *cyp3a65*, *cyp2k18*, *ugt1a1*, *abcc2*) suggests enhanced xenobiotic detoxification, while interactions with *cyp7a1* and *lcat* indicate potential effects on lipid homeostasis. These findings imply that TnP may alter the pharmacokinetics of co-administered drugs, calling for caution in clinical applications to avoid drug-drug interactions.

Notably, TnP also influences the redox balance (*gpx3*, *gstt1b*) and immune modulation (*ido1*), linking drug metabolism to inflammatory responses. The dual regulation of metabolic activation (CYPs) and suppression (fatty acid metabolism genes) suggests a complex, context-dependent mechanism that warrants further investigation.

TnP might accelerate tissue repair by modulating muscle contraction (*myhb*, *mylz3*, *myha*), innate immunity via *tlr5*-*cdc42* and mucin-chemokine axes (*tlr5a/b*, *cxcr3.3*, *ifngr1*), and neuro-immune crosstalk (*gabrd*, *drd1b*, *ednrba*). The *tlr5*-*cdc42* axis suggests that TnP enhances pathogen recognition and leukocyte recruitment, while interactions between cadherins and GTPases (*cdhr2*, *cdc42ep1a*) indicate roles in cell adhesion and migration. Additionally, mucin-chemokine interactions (*muc5.1*, *vwa11*) suggest modulation of the mucosal barrier. These findings position TnP as a potential therapeutic for chronic wounds, enabling synchronized progression through inflammatory to resolution phases; however, risks such as hypercontractility or excessive inflammation must be considered.

TnP activates a protease network (*c6ast4*, *cpa1/4/5*, *prss59.1*) that facilitates ECM remodeling, which is crucial for wound healing and fibrosis resolution. Integrating autophagy (*atg2a*, *ulk1a*) and metabolic signaling (*g6pca.1*, thyroglobulin) indicates that TnP optimizes energy utilization during tissue repair. Key regulatory nodes include *bcl6* paralogs, which suppress proteases and metabolic genes (*ela2*, *lct*) and phosphatases (*ppp3ccb*, *ppp6r2b*), which modulate muscle energetics and autophagy. This system-wide coordination highlights TnP’s potential in fibrosis treatment and metabolic disorders, though excessive protease activity could lead to tissue damage.

TnP influences melanogenesis (*pmela*/*pmelb*, *dct*, *tyr*) and circadian protein turnover (*fbxl3l*/*fbxl8*), linking pigmentation to ubiquitin-dependent degradation. It also modulates lipid metabolism (*ucp3*, *lpin1*, *pnpla2*) and desmosome integrity (*klhl24b*), suggesting roles in skin barrier function and metabolic diseases. The short path lengths in this network indicate rapid, synchronized regulation, making TnP a candidate for pigmentary disorders (e.g., vitiligo) and circadian dysfunction.

## 4. Materials and Methods

### 4.1. TnP Peptide

TnP trifluoroacetate compound (C_63_H_114_N_22_O_13_S_4_, 97.3% purity, IPR[CRKMPGVKMC]-NH2, MW 1516.00, pI 10.63) synthesized in the solid phase was purchased from GenScripts (#P13821401; Piscataway, NJ, USA) and diluted in 1× E3 embryo medium (5 mM NaCl, 0.17 mM KCl, 0.33 mM CaCl, and 0.33 mM MgSO_4_ dissolved in MilliQ water, pH 7.0).

### 4.2. Zebrafish Injury Model and TnP Treatment

Fertilized eggs of wild-type AB zebrafish were provided by the Karolinska Institutet Zebrafish Core Facility (Solna, Sweden) and raised until 48 h post fertilization (hpf) at 28 °C in 1× E3 embryo medium. For the treatment, a working solution of 100 mM TnP or ultra-pure water as a control was injected (2–4 nL) into the yolk of manually dechorionated embryos. For manipulation, embryos were anesthetized with tricaine methane sulfonate at 0.02% (#MS-222, Sigma Aldrich, St. Louis, MO, USA). One day after the TnP treatment, at 72 hpf, an injury-induced inflammation model was established in the larvae by tail fin amputation, as described in Disner et al. [21]. The caudal fin was chosen for investigation because its characteristics allow for the study of the process of early sterile inflammatory response, and it is the largest of the zebrafish fins, simplifying manipulation and observation [29]. Larvae were kept in standard raising conditions for recovery and to mount the inflammatory response for two hours. Then, larvae were sampled (10 per group; quadruplicate) in RNAlater Stabilization Solution (Invitrogen, Carlsbad, CA, USA) before further processing. The experimental procedures adhered to ethical principles and complied with strict animal welfare guidelines. They were conducted in accordance with the EU Directive 2010/63/EU on the protection of animals used for scientific purposes.

### 4.3. RNA Purification and Sequencing

RNA purification was performed using the RNeasy Plus Universal Mini Kit (Qiagen, Hilden, Germany; #73404) following the manufacturer’s instructions. mRNA purification, cDNA synthesis, and clustering were performed as described in Disner et al. [21]. The library preparation was sequenced on a state-of-the-art Illumina NovaSeq platform using a short-read sequencing strategy, generating 150 bp paired-end reads.

### 4.4. Data Analysis

Cleaned data were mapped to the *Danio rerio* reference transcriptome provided by Ensembl GRCz11 V.109, and the transcripts were quantified with the Salmon tool, version 0.8.2 [30]. Then, the gene expression matrix was constructed using quantified transcripts with the R/Bioconductor Tximeta package, version 1.0.3 [31]. We used the ensemble packages org.Dr.eg.db to annotate the quantified transcripts according to V.109 of the reference ensemble used for quantification [32,33].

For differential expression (DE) analysis, counts from the filtered gene expression matrix, limited to protein-coding genes, were used. Genes with a total count of less than 10 across all samples and replicates were removed. The compared groups involving induced inflammation and TnP treatment underwent DE analysis using the R/Bioconductor DESeq2 package [34]. The *p*-values were calculated using the Wald test and adjusted via the Benjamini–Hochberg approach to control the false-discovery rate. Genes with an adjusted *p*-value (*p*_adj_) ≤ 0.05 were identified as differentially expressed genes (DEGs). A log2 fold change (FC) threshold of ± 0.5 was also applied for significance (DEGs = *p*_adj_ < 0.05, log2FC ± 0.5).

Protein-protein interactions network of upregulated DEGs identified in the study was performed in STRING on Cytoscape v3.10.3 [35] for the following expert-driven categories: (I) drug metabolizing enzymes, (II) cellular traffic, (III) cell activity, and (IV) signaling pathways. STRING is an established tool for interpreting and reducing newly acquired genetic screening datasets, and its link to functional associations helps to suggest evidence of a specific partnership between two proteins [36]. In addition, functional enrichment analysis was conducted using DAVID software v2023q4 [37], where enriched terms were considered significant if the adjusted *p*-value was less than 0.05 and the gene count at least 3.

## 5. Conclusions

In conclusion, TnP emerges as an integrated modulator with therapeutic potential, most notably in the resolution of inflammation, wound healing, and drug metabolism, and to a lesser extent in metabolic and pigmentary disorders. However, its broad effects demand careful consideration while dosing to avoid off-target impacts (e.g., DDIs, excessive proteolysis). Other limitations include the lack of dose comparisons and the exploratory nature of this study, which was primarily based on global gene expression profiles, potentially reducing organ or tissue specificity. Additionally, although the expert-driven categorization of the DEGs into functional groups helps narrow down the uncovered therapeutic effects of the candidate peptide, one must consider the multiple roles of the genes, which can participate concurrently in different pathways. Moreover, the levels of expression do not necessarily reflect the corresponding proteins due to regulatory checkpoints, and the functional interpretation based on enrichment analysis may be limited by the available genomic annotation for the zebrafish model. Future studies should validate TnP’s effects in mammalian models to assess translational potential, investigate tissue-specific delivery to minimize systemic side effects, and explore combinatorial therapies (e.g., with anti-fibrotic or metabolic drugs).

This study integrated gene expression profiling and protein-protein interaction networks to characterize the multi-target mechanisms of TnP systematically. Network proximity analysis revealed that TnP significantly modulates key biological processes, including Phase I drug metabolism and peptide transport, tissue repair, immune regulation, and neuro-metabolic crosstalk. These findings highlight the broad therapeutic potential of TnP for inflammatory/autoimmune diseases, neuromuscular disorders, and neuroendocrine or neurodegenerative conditions. Overall, TnP represents a novel multi-pathway therapeutic agent, but its clinical application requires careful optimization to harness its benefits while mitigating risks.

## Figures and Tables

**Table 1:** List of the genes included in the network analysis performed by STRING on Cytoscape in each of the corresponding categories.

Likely Biological Function	Gene Symbol	Gene Name
Drug-metabolizing enzymes	*ido1*	Indoleamine 2,3-dioxygenase 1
	*gstt1b*	Glutathione S-transferase theta 1b
	*gpx3*	Glutathione peroxidase 3
	*ugt5c3*	UDP glucuronosyltransferase 5 family, polypeptide C3
	*ces2b*	Carboxylesterase 2b
	*ugt5a2*	UDP glucuronosyltransferase 5 family, polypeptide A2
	*cyp1a*	Cytochrome P450, family 1, subfamily A
	*abcc2*	ATP-binding cassette, sub-family C (CFTR/MRP), member 2
	*cyp2k21*	Cytochrome P450, family 2, subfamily k, polypeptide 21
	*hsd11b1lb*	Hydroxysteroid (11-beta) dehydrogenase 1-like b
	*ugt1a1*	UDP glucuronosyltransferase 1 family, polypeptide A1
	*cyp3a65*	Cytochrome P450, family 3, subfamily A, polypeptide 65
	*cyp2p6*	Cytochrome P450, family 2, subfamily P, polypeptide 6
	*cyp2n13*	Cytochrome P450, family 2, subfamily N, polypeptide 13
	*cyp2v1*	Cytochrome P450, family 2, subfamily V, polypeptide 1
	*cyp2k18*	Cytochrome P450, family 2, subfamily K, polypeptide 18
	*cyp3c3*	Cytochrome P450, family 3, subfamily c, polypeptide 3
	*cyp2aa11*	Cytochrome P450, family 2, subfamily AA, polypeptide 11
	*cyp4v7*	Cytochrome P450, family 4, subfamily V, member 2b
	*slc7a7*	Solute carrier family 7 member 7
	*slc15a1b*	Solute carrier family 15 member 1b
	*slc6a15*	Solute carrier family 6 member 15
	*slc43a2a*	Solute carrier family 43 member 2
	*slc6a19b*	Solute carrier family 6 member 19b
	*cyp7a1*	Cytochrome P450, family 7, subfamily A, polypeptide 1
	*abca1a*	ATP-binding cassette, sub-family A (ABC1), member 1A
	*abcg1*	ATP-binding cassette, sub-family G (WHITE), member 1
	*lratb.2*	Lecithin retinol acyltransferase b, tandem duplicate 2
	*rpe65a*	Retinoid isomerohydrolase RPE65 a
	*rgra*	Retinal G protein coupled receptor a
	*slc20a2*	Solute carrier family 20 member 2
	*slc34a2b*	Solute carrier family 34 member 2b
	*slc15a4*	Solute carrier family 15 member 4
	*slc26a3.2*	Solute carrier family 26 member 3, tandem duplicate 2
	*cytb*	Cytochrome b, mitochondrial
	*znf704*	Zinc finger protein 704
	*slc16a6b*	Solute carrier family 16 member 6b
	*slc16a9a*	Solute carrier family 16 member 9a
	*lcat*	Lecithin-cholesterol acyltransferase
	*mogat2*	Monoacylglycerol O-acyltransferase 2
	*smpdl3b*	Sphingomyelin phosphodiesterase acid like 3B
	*gpcpd1*	Glycerophosphocholine phosphodiesterase 1
	*pdk4*	Pyruvate dehydrogenase kinase, isozyme 4
	*pdk2a*	Pyruvate dehydrogenase kinase 2a
	*abcb11b*	ATP binding cassette subfamily B member 11
	*slc10a2*	Solute carrier family 10 member 2
	*cyp24a1*	Cytochrome P450, family 24, subfamily A, polypeptide 1
	*slc8a1b*	Solute carrier family 8 member 1b
Cellular traffic	*myhb*	Myosin, heavy chain b
	*mylz3*	Myosin, light polypeptide 3, skeletal muscle
	*myha*	Myosin, heavy chain a
	*mybpc2a*	Myosin binding protein Ca
	*tmem184ba*	Transmembrane protein 184ba
	*gabarapb*	GABA(A) receptor-associated protein b
	*urgcp*	Upregulator of cell proliferation
	*ednrba*	Endothelin receptor Ba
	*vwa11*	Von Willebrand factor A domain containing 11
	*cxcr3.3*	Chemokine (C-X-C motif) receptor 3, tandem duplicate 3
	*tlr5a*	Toll-like receptor 5a
	*tlr5b*	Toll-like receptor 5b
	*muc5.1*	Mucin 5.1, oligomeric mucus/gel-forming
	*cdhr2*	Cadherin related family member 2
	*cdhr5b*	Cadherin-related family member 5b
	*LOC568392*	Cadherin-1-like
	*cdc42ep1a*	CDC42 effector protein (Rho GTPase binding) 1a
	*nhsa*	NHS actin remodeling regulator
	*gabrd*	Gamma-aminobutyric acid type A receptor subunit delta
	*drd1b*	Dopamine receptor D1b
	*ifngr1*	Interferon gamma receptor 1
	*crfb16*	Cytokine receptor family member B16
Cell activity	*c6ast4*	Six-cysteine containing astacin protease 4
	*prss59.1*	Serine protease 59, tandem duplicate 1
	*prss1*	Serine protease 1
	*cpa1*	Carboxypeptidase A1 (pancreatic)
	*cpa5*	Carboxypeptidase A5
	*serpinf2a*	Serpin peptidase inhibitor, clade F (alpha-2 antiplasmin, pigment epithelium derived factor), member 2a
	*cpb1*	Carboxypeptidase B1
	*cela1.4*	Chymotrypsin like elastase family member 1, tandem duplicate 4
	*cel.1*	Carboxyl ester lipase, tandem duplicate 1
	*pdia2*	Protein disulfide isomerase family A, member 2
	*amy2a*	Amylase alpha 2A-like 2
	*meltf*	Melanotransferrin
	*cel.2*	Carboxyl ester lipase, tandem duplicate 2
	*ela2*	Elastase 2
	*cpa4*	Carboxypeptidase A4
	*atg2a*	Autophagy related 2A
	*ulk1a*	Unc-51 like autophagy activating kinase 1a
	*mlst8*	MTOR associated protein, LST8 homolog (*S. cerevisiae*)
	*anpepb*	Alanyl (membrane) aminopeptidase b
	*ace2*	Angiotensin I converting enzyme 2
	*bcl6ab*	BCL6A transcription repressor b
	*bcl6aa*	BCL6A transcription repressor a
	*lct*	Lactase
	*g6pca.1*	Glucose-6-phosphatase catalytic subunit 1a, tandem duplicate 1
	*mb*	Myoglobin
	*tg*	Thyroglobulin
	*ppp6r2b*	Protein phosphatase 6, regulatory subunit 2b
	*ppp3ccb*	Protein phosphatase 3, catalytic subunit, gamma isozyme, b
Signaling pathways	*pmelb*	Premelanosome protein b
	*dct*	Dopachrome tautomerase
	*tyr*	Tyrosinase
	*pmela*	Premelanosome protein a
	*fbxl3l*	F-box and leucine-rich repeat protein 3, like
	*fbxl8*	F-box and leucine-rich repeat protein 8
	*LOC100004499*	Stereocilin
	*chs1*	Chitin synthase 1
	*chia.2*	Chitinase, acidic.2
	*chia.1*	Chitinase, acidic.1
	*ucp3*	Uncoupling protein 3
	*lpin1*	Lipin 1a
	*pnpla2*	Patatin-like phospholipase domain containing 2
	*crebrf*	Creb3 regulatory factor
	*klhl24b*	Kelch like family member 24
	*histh1l*	Histone H1 like1
	*h1-10*	H1.10 linker histone

## Data Availability

Data is contained in the paper or available from the corresponding author upon request.

## Supplementary Materials

The following supporting information can be downloaded at: https://www.mdpi.com/article/10.3390/ph18081146/s1; Table S1: Differentially expressed genes classified into four cathegories: I—Drug metabolizing enzymes, II—Cellular traffic, III—Cell Activity, and IV—Signaling pathways.

## Abbreviations

ADME	Absorption, Distribution, Metabolism, and Excretion
AHR	Aryl Hydrocarbon Receptor
CNS	Central Nervous System
CYP	Cytochrome P450
DDI	Drug–Drug Interaction
DE	Differential Expression
DEG	Differentially Expressed Gene
DME	Drug-Metabolizing Enzyme
EAE	Experimental Autoimmune Encephalomyelitis
ECM	Extracellular Matrix
FC	Fold Change
GABA	Gamma-Aminobutyric Acid
GST	Glutathione S-Transferase
GTPase	Guanosine Triphosphatase
HPF	Hours Post Fertilization
HPI	Hours Post Injury
LPS	Lipopolysaccharide
MS	Multiple Sclerosis
mTOR	Mechanistic Target of Rapamycin
NAT	N-acetyltransferases
PPI	Protein–Protein Interaction
RNA	Ribonucleic Acid
SLC	Solute Carrier
SULT	Sulfotransferase
TLR	Toll-Like Receptor
UGT	UDP-Glucuronosyltransferase
